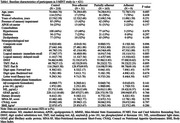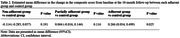# The influence of the adherence on intervention effect: J‐MINT study

**DOI:** 10.1002/alz.088996

**Published:** 2025-01-09

**Authors:** Kazuaki Uchida, Taiki Sugimoto, Nanae Matsumoto, Kosuke Fujita, Yoko Yokoyama, Yujiro Kuroda, Takashi Sakurai, Hidenori Arai

**Affiliations:** ^1^ National Center for Geriatrics and Gerontology, Obu, Aichi Japan; ^2^ University of Washington, Seattle, WA USA

## Abstract

**Background:**

This study aimed to examine the influence of adherence on intervention effect in the Japan‐multimodal intervention trial for the prevention of dementia (J‐MINT).

**Method:**

J‐MINT was an 18‐month randomized controlled trial, and participants aged 65‐85 years with mild cognitive deficits were randomized into multidomain intervention (physical exercise, nutritional counseling, cognitive training, and vascular risk factor management) and control groups. This study included the participants in J‐MINT who had undergone the intervention program and follow‐up evaluation at least once. Adherence in each intervention component was classified as adherent (1 point) or non‐adherent (0 point), and the total score was calculated (score range: 0‐4 points). Adherent was defined as follows: the adherence rate to group‐based physical exercise ≥ 70%, the number of nutritional counseling ≥ 14 times, the number of days of cognitive training ≥ 156 days, and those who have blood pressure, glucose levels, and cholesterol within the target range. Three groups were created based on the total score: adherent group (4 points), partially adherent group (2‐3 points), and non‐adherent group (0‐1 points). The outcome was the changes in composite score calculated by eight neuropsychological tests from baseline to 18 months, including tests of global cognitive function, memory, attention, and executive function/processing speed. Mixed models for repeated measures were used to calculate mean differences (MD) of outcome between each adherence and control group.

**Result:**

Out of 531 participants enrolled in J‐MINT, 421 participants (mean age: 73.8 years, 52% female) were included. The number of participants in the adherence group was 9 in the adherent group, 163 in the partially‐adherent group, and 31 in the non‐adherent group. Statistical analysis showed that the adherent group exhibited a significant interventional effect compared to the control group (MD = 0.266, p = 0.025), while no significant mean difference was observed between the other adherence group and the control group.

**Conclusion:**

Our findings suggest that supporting adherence is important to achieve the sufficient effect of multidomain interventions.